# Address to Graduates of the Philadelphia Dental College

**Published:** 1884-04

**Authors:** S. H. Guilford


### Independent Practitioner.

VOL. V. APRIL, 1884. No. 4.

### Original Communications.

### ADDRESS.

TO GRADUATES OF THE PHILADELPHIA DENTAL COLLEGE AT THE TWENTY-FIRST COMMENCEMENT, ACADEMY OF MUSIC, PHILADELPHIA, FEB. 29, 1884.

BY PROF. S. H. GUILFORD, A. M., D. D. S.

*Gentlemen of the Graduating Class:*

The last act of your student life has been performed, and at the hands of the President of the Board of Trustees you have received your diplomas, as evidences of your fitness and qualification to practice the profession of your choice. The faculty of the Philadelphia Dental College have selected me as their representative to tender you their congratulations (warm as heart can wish), upon the successful completion of your college course.

In the years you have been with us your powers of body and mind have both been sorely taxed by your arduous daily duties, but through it all you have been supported and encouraged by the hope that when this time should come, you might be accounted worthy to stand upon this stage and receive the evidence of your proficiency. Your hopes have been realized, and we rejoice with you in your success. The distinction that existed between us as teacher and pupil is now removed, and we stand upon one common level. As practitioners of dentistry we welcome you to our ranks.

Yesterday you were students; to-night you go out from among us to enter the arena of public life, there to win such success as may lie in your power to attain.

The change, gentlemen, from student life to active practice, will be a great one. Up to this point in your studies and in your work you have had constantly by your side those who could and did advise you when you needed help. Now you will be thrown upon your own responsibility and resources, and must stand alone. Your own judgment must guide you, and your own skill be your support. Your lot will be a hard one, as it always is in the earlier years of a man's professional life, and yet, as others have gone through the struggle successfully, why may not you? As you stand here to-night, on the threshold of your professional career, before we extend the hand of parting and you go out from under the parental roof, it may not be amiss to impress upon you some truths and facts that may aid you in your career.

Each one of you starts out with a fixed determination to be successful in life. But, gentlemen, let me ask you, what do you mean by success, and what idea does the word convey to your mind? Is it the idea of the vulgar world that success means the gathering of money; or is it that higher and nobler idea that success is measured by the amount of good done?

The man who enters professional life for the sole purpose of gathering riches, recognizing nothing nobler and higher in it, debases his profession and prostitutes himself. His object is ignoble, and his methods will surely be base. Such an one, and their number is great, will turn back the wheels of professional progress in a twelvemonth more than they can be advanced in a decade. The world would be better if he had never been born. It is the philanthropic or humanitarian element in professional life that helps to distinguish it from the purely mercantile, and he who eliminates this element from it changes its character and destroys its identity.

True, money-getting is a right and proper motive when properly controlled, but the professional man who makes it his chief or only object utterly fails to appreciate the true intent of his vocation. Be assured, gentlemen, that professional success consists in the measure of good we do unto others; in the amount of suffering we alleviate, and the degree of happiness and comfort we are capable of administering. Do not fear that in thus carrying out the lofty and true ideal you will suffer, for philanthropy, like charity, is twice blessed, in that it blesses him that gives as well as him that doth receive. He who works up to the highest ideal will have his efforts appreciated, and money and fame, besides peace of conscience, will be added unto him.

But you ask, how shall we attain this success? The conditions of it, gentlemen, are easily understood. The principles that underlie it in your vocation are the same as those that condition it in every department of life.

First, you must impress yourselves with the idea that your calling is the greatest of all callings. Unless you properly appreciate the importance of it, you will never bring out the best that is in it. The enthusiast in his work, whatever that work may be, is the one who usually meets with the greatest measure of success.

Next, it is necessary that you possess the requisite knowledge and skill to enable you to perform your professional duties in the best and most acceptable manner. This ability, or so much of it as you are able to obtain outside of the hard school of experience, we believe you to possess. To obtain it you have passed through your long period of pupilage; your studies have covered the widest range; your clinics have been of the most varied and comprehensive character, and the opportunities to put into actual practice the methods taught have been the greatest we could give you.

The last condition is untiring industry. Well-directed effort is one of the very largest factors in the equation of success. Success is rarely, if ever, attained without it, no matter how great the natural ability, whilst with it, even mediocrity rises to a high plane and secures a commanding position. Hard work, gentlemen, is the great thing that tells in life.

Many, and young men especially, are apt to attribute the success of men who have attained a high position in life to their possession of genius, or some special qualities denied to the majority, apparently forgetting that—

> " The heights by great men won and kept
> Were not attained by sudden flight;
> But they, while others peaceful slept,
> Toiled slowly upward in the night."

Activity is the great need of the day, and the lack of it is almost sure to result in failure.

The great and absorbing idea of most young men on starting out in professional life, is to secure a practice and gain a competency. This, of course, is your first need, but practices come slowly. Few men start in with much to do. The world, when you begin, does not need you half as much as you thought it did. In the earlier years of your practice you will have more unoccupied hours than busy ones. How these spare hours are spent often determines the future success or failure of the individual. Spent in idleness and useless repinings over not having more to do, they will be to you a source of great unhappiness and discontent; but spent in useful activity, they will be of the greatest possible advantage to you.

Ah! gentlemen, these spare hours in the beginning of your practice, that you are apt so greatly to deplore, are hours of golden opportunity. True, you cannot turn them to immediate money account, but they will bring you a good return in the end if you but rightly employ them. Be wise, and learn this in time.

Do you ask me how you shall employ them? I answer—use them for the pursuit of knowledge. If you have employed your time to the very best advantage thus far, what you now know is but a fraction of what you may know. We all feel justly proud of the advance in knowledge made by our profession in the last fifty years, but there is much still that we do not know, and that we are anxious to learn. You have it in your power to shed light upon much that is at present shrouded in darkness, if you will but make the effort. Your spare hours in early practice will afford you the opportunity, and chemistry and microscopy will afford you the means. These two helps will prove the best friends for your advancement in knowledge and fame that you can possibly have. Use them well and use them diligently, and the full effect of their influence will only be fully realized later on in life.

They will enable you, as they have others, to penetrate the mysteries of life and being, to know the causes of existing conditions, and open the way to the discovery of their remedies. Forty years ago a student of medicine at the College of France was presented by a fellow student with a microscope which he no longer needed. The young man knew nothing of the construction of the instrument, and very little of its use. He felt no need of it, but, offered as a present, he could not well decline its acceptance. He studied, first its construction, and then set about to learn how to use it. A remark that he one day overheard, made by a quack physician, as to his idea of the origin and transmission of disease, led him into a series of examinations and experiments, which, continued unremittingly for a long time, led to the establishment and elucidation of the germ theory of disease. The young man that then was, is now M. Pasteur the distinguished French scientist, member of the French Academy, and one whose name is honored wherever the name of science is known.

On the cover of one of our journals you will notice the inscription, "Observe, Compare, Reflect, Record." Take this as your motto, and let its precepts be your guide in the pursuit of knowledge.

It is one thing to see, and quite another to observe. Many do the former, few the latter. Observation carries with it in its meaning a seeing closely, and with such exactness as to leave a strong impress upon the memory. Many an important fact or condition in scientific or professional life is unnoticed or passed by through lack of careful observation. Close observation is one of the foundation stones upon which the structure of science is reared. Cultivate it, therefore, for without it you build in vain. Things that seem trifling to us at the time may, and often do, become of immense moment to us in the course of years.

Toward the close of the last century, two young students at the French military school of St. Etienne happened to be sailing on a river near by. One of them, with one hand on the tiller and the other grasping the main-sheet, was sailing the boat in its zigzag course against the wind. The other was occupied in lazily paying out and hauling in a long cord that he held in his hand. He who was sailing the boat reproved his companion for his listlessness, and begged him desist from his idle play. The other refused, saying that he had set out to determine the width of the stream by triangulation and measuring the length of the vessel's course, and he was bound to complete it.

It seemed like an idle fancy to spend his time thus, but twenty years later when he appeared upon the same scene as Napoleon Bonaparte, and planted his cannon upon one bank of the river, his knowledge of the exact width of the stream enabled him to so sight his guns as to speedily reduce the fort on the opposite bank that had been attempting to impede his progress.

Observation, however, in your own line of work, will not be sufficient to accomplish the end you have in view. It must be supplemented by the observation of others. What they have done you will find in the literature of the profession, in its text books and periodicals. Consult them and study them well. The result of no one man's work is sufficient to establish important truths. Each one is liable to err in his researches and deductions therefrom, and it is only by a careful comparison of our own investigations with those of others that errors can be eliminated and truths established. But what you arrive at in this way you must not trust to memory to retain. Memory is a fickle faculty, and we dare not depend too much upon it. If it be important to ascertain truths, it is quite as important to preserve them. This can only be done by systematically recording them, so that they may be preserved to you and may benefit others. What you have learned in your studies and attendance upon lectures has not been drawn from the knowledge of a half dozen men, but represents the wisdom and learning of all those who have preceded you in professional life, culled for your benefit, and presented in a form to be received and appreciated by you. In thus accepting the combined results of those who have gone before you, you are placed under a virtual obligation to contribute your portion of knowledge for the benefit of those who come after you. See that you discharge this obligation with fidelity. Your first duty is toward yourselves, but after that comes your duty toward others—a giving, as it were, in return for what you have received. Such contribution, on your part, to the general fund of knowledge, while it benefits others, will not be without reflex influence upon yourselves. In proportion as you gain knowledge and give it to the world will you receive honor in the eyes of your associates. Your knowledge and diligence, added to your trained habits of study and observation, will make you valuable, and lead to your being placed upon the list from which the leaders of the future are to be chosen.

Be ambitious, therefore, to excel, and strain every nerve to play well your part upon the theatre of the world's action. Those who now occupy the places of professional preferment will, in a few short years, be obliged to give place to other and younger men, and when that time comes those best fitted will be the ones selected.

That great financial institution, the Bank of England, is governed by twenty-four directors. They are chosen for life, and their position is one of envied prominence and responsibility. As each one dies or retires his place is filled by a young man, so that he may grow up and become thoroughly identified with the workings and interests of the institution. The choice of a successor seems in each case to be quickly made, but it is not so in reality. The many young men of business in London, unconsciously to themselves, are being constantly watched, for from their number must come the one to fill the next vacancy that may occur in the directorship. Their diligence, their honesty, their business capacity are all noted, and the selection for the next place is secretly made long before the necessity for it exists.

So, gentlemen, do not imagine that as you go out from the institution to-night you go out to be forgotten or lost sight of. The eyes of your old teachers and those who know you will follow you and rest upon you. Your course in life will be closely watched. If you do well we will all rejoice in it, and if you do ill our hearts will be filled with sorrow. We all love our profession and wish for its advancement; but remember that a profession can only be advanced in proportion to the advancement of its members.

To-night has initiated you into such membership; see that you prove yourselves worthy of it. Remember your duties to your profession, and remember also your duty to your Alma Mater. What credit you gain in life will in a measure be reflected upon her; whereas, if lack of honor should be your portion, she will have to suffer with you.

Of the many hundred graduates who have gone forth from our institution in the twenty-one years of its existence, a large proportion have risen to positions of prominence in their calling. Will you add to the number? As I look into your upturned faces to-night, many of whom we may not see again until time has left its mark upon them, I am reminded more than ever of the cosmopolitan character of those who year after year comprise our classes. Our own country is represented here by States bounded only by Maine and Texas, and the Atlantic and Pacific coasts. Canada and Nova Scotia, from the far north, are here, and tropical Mexico has her representative. From across the ocean Prussia, and Germany, and England, and Scotland have sent their sons, and even the isles of the sea, sunny Cuba, and far-off Australia are here represented. When you came to us from so many lands you came as strangers. The ties that at first bound us together, attenuated as they were, have, through long and intimate communion, grown to strong cords, and we find them hard to sever. Still, hard as it is to sunder our relations as teacher and pupil, it must be done. You must needs go out to do the work that awaits you, and others are waiting to take your place. We bid you go then, and God-speed, for if you will but live up to the measure of your abilities the world has need of you. Be filled with enthusiasm for your work, and be guided by a laudable ambition, remembering meanwhile that—

> "Not without toil is fame's bright palace won,
> Nor glory's race with faltering footsteps run.
> The richest fruit the highest bough adorns;
> The lovliest rose is guarded most by thorns.
> Deep in the ocean precious pearls do shine;
> The brightest diamond seeks the darkest mine;
> And that which is with greatest toil possessed
> We prize the longest and we love the best."

